# A decision-tree for trend analyses following major survey redesigns: an analysis using the Canadian Community Health Survey (CCHS)

**DOI:** 10.1186/s12889-026-28851-7

**Published:** 2026-08-08

**Authors:** Alexandra Blair, Matthew E. Perks, Rabina Jahan, Eric Vallières, Le Li, Dolon Chakravartty, Beth Jackson

**Affiliations:** 1 Public Health Agency of Canada, Health Equity Division, Ottawa, Canada; 2 University of Toronto, Dalla Lana School of Public Health, Toronto, Canada; 3 York University, School of Health Policy and Management, Toronto, Canada

**Keywords:** Canada, Population Health, Surveillance, Survey, Bias, Mode Effects

## Abstract

**Background:**

Monitoring health inequality trends is essential for assessing progress, stagnation, or regression on the reduction of health inequalities; assessing the impact of certain policies on health inequalities; and identifying emerging areas of concern. Because inequalities can change slowly, long-term data (≥ 15 years) may be required to detect meaningful shifts. However, methodological changes in data sources over time raise concerns about data comparability. Frameworks are needed to guide decisions on whether data should be combined in these instances.

**Methods:**

We used the Canadian Community Health Survey (CCHS) as a case to explore how sensitivity analyses and a decision-tree could help assess the comparability of survey cycles before and after major CCHS redesigns in 2015 and 2022, balancing considerations of statistical and practical significance, and background trends.

Using CCHS cycles 2007–2008 to 2024, we estimated crude prevalence of 20 health indicators across cycles, among adults. We assessed: 1) the relative percent (%) change and 2) absolute percentage-point (PP) change between cycles pre- and post-redesigns (i.e., 2013–2014 vs. 2015–2016, 2019–2021 vs. 2022), including tests of statistical significance. Then, using generalized linear models and their predicted probabilities, we modeled the predicted post-redesign prevalence (for 2015–2016, 2022), based on pre-redesign trends (2007–2014, 2015–2021, respectively). Drawing from literature-based thresholds, the proposed decision-tree was used to flag indicators that met the three criteria described below. These indicators were subjected to additional qualitative investigation. Specifically, the flagged indicators must have demonstrated practically significant change on: A) relative (≥ 10% change) and B) absolute scales (≥ 2.5- or 5-PP change, based on baseline prevalence); and C) statistically significant change (no overlap with the margin of equivalence (95% CI) between observed and predicted post-redesign prevalence).

**Results:**

No indicators met all three criteria post-2015. Post-2022, food insecurity, and self-rated physical and mental health marginally met all three criteria, signaling a need for additional research for long-term reporting.

**Conclusions:**

This study presents a novel and reproducible framework for CCHS-specific analyses that others can refine or adapt. As it remains sensitive to assumptions, caution notes regarding the redesigns continue to be warranted for all CCHS indicators.

**Supplementary Information:**

The online version contains supplementary material available at https://doi.org/10.1186/s12889-026-28851-7.

## Introduction

Examining trends in health inequalities is essential for the monitoring of current and emerging priorities and inequalities in public health. However, the feasibility of trend analyses is dependent on several factors, namely data availability and comparability over time. The Public Health Agency of Canada (PHAC) is responsible for the Pan-Canadian Health Inequalities Reporting Initiative (HIRI), which measures and monitors trends in health inequalities in Canada. A primary data source for HIRI is the Canadian Community Health Survey (CCHS), which underwent major redesigns in 2015 and 2022 [1]. In 2015, changes included a review and refinement of sampling methodology, sample frame, and adjustments to content [1]. In 2022, changes were made to collection and sampling approaches, including a transition to an electronic questionnaire for direct self-reporting, which can introduce “mode effects” on reported health outcomes [1] (changes are described in detail in Supplemental eTable 1). These changes potentially undermine the validity of comparing CCHS cycles before and after 2015 and 2022, in HIRI’s analyses of trends in inequalities over time.

CCHS custodian Statistics Canada advises that “as a result of these redesigns and major changes to collection and sampling approaches as well as content updates, caution should be taken when comparing data from previous cycles to data released for 2015 and 2021 and for data released 2022 and onwards” [1]. Nonetheless, several research projects and government reports continue to combine data before and after these redesigns (examples are described in Supplemental eTables 2 and 3). However, there is a lack of guidance and documented examples of how researchers determined whether cycles before and after these redesigns should be compared, particularly in a health inequality monitoring context.

When deciding whether to compare data across redesigns, one important consideration is whether there was a meaningful change in the estimated prevalence of an outcome after a redesign took place. Several strategies have been employed in public health and epidemiologic literature to assess meaningful changes in prevalence between two time points or groups of people. Two key dimensions of these strategies are the statistical and practical significance of the changes [2]. Here, “practical significance” refers to changes that are meaningful in the “real-world” (e.g. in public health or health policy) settings [3]. Additionally, one must consider whether the assessment of practically significant change is assessed on relative and/or absolute scales [4–6]. There are few frameworks available that describe how the assessment of relative and absolute scales should be jointly considered when deciding whether survey cycles are sufficiently comparable to be used in analyses.

To address this gap, this study explores sensitivity analyses that can be used to assess the validity of comparing survey cycles before and after major survey redesigns, while considering pre-existing background trends. It proposes a decision-tree that balances considerations of statistical and practical significance. It is not intended to represent a definitive guideline, but rather a transparent, reproducible example that fills a gap in Canadian epidemiologic literature, and that others may further test, adapt or build upon.

## Methods

### Data and study population

We used data from cycles 2007–2008 to 2024 of the CCHS, which is a nationally representative cross-sectional health and social survey of Canadians [1]. Although these cycles surveyed respondents aged 12 and above, our analyses were restricted to respondents aged 18 years and above to ensure consistency in the interpretability of outcome prevalence in the adult population over time. Available combined cycles (e.g. 2007–2008 to 2019–2020) were used, when possible, to ensure both provincial and territorial data coverage (additional details are provided in Supplemental eTable 4). Data from the Yukon, the Northwest Territories and Nunavut were available in all cycles except 2021, 2023, 2024. Some indicators had uneven provincial coverage in certain cycles (eTable 4). In these situations, national estimates were calculated using all available provincial and territorial data for each period.

Populations excluded from all CCHS cycles include: persons living in institutionalized settings (e.g., hospital, nursing home, live-in work arrangement, prison or in custody) [7], on reserves and other First Nations settlements in the provinces; full-time members of the Canadian Forces; and persons living in the Quebec health regions of Région du Nunavik and Région des Terres-Cries-de-la-Baie-James. Altogether, these exclusions represent less than 3% of the Canadian population [1].

### Measures

Twenty HIRI indicators were explored in this study. All were coded as binary variables (presence versus absence of the outcome). The indicators included self-reported anxiety, arthritis, asthma, chronic obstructive pulmonary disease (COPD), diabetes (excluding gestational diabetes), diabetes (including gestational diabetes), moderate or severe food insecurity (vs. no food insecurity), heart disease, hypertension, high perceived life stress (defined as reporting most days as “quite a bit” or “extremely” stressful vs. “not at all”, “not very”, or “a bit” stressful), high life satisfaction (defined as being “satisfied” or “very satisfied” vs. “neither satisfied nor dissatisfied”, “dissatisfied”, or “very dissatisfied”), mood disorder, obesity, fair or poor self-rated mental health (vs. “good”, “very good”, or “excellent”), very good or excellent self-rated mental health (vs. “good”, “fair”, or “poor”), fair or poor self-rated health (vs. “good”, “very good”, or “excellent”), very good or excellent self-rated health (vs. “good”, “fair”, or “poor”), smoking (daily or occasionally), exposure to second hand smoke in the home, strong or very strong sense of community belonging (vs. “somewhat weak” or “very weak”). Aligned with Canadian public health stakeholder interests, particularly for both strengths- and deficit-based health monitoring, HIRI releases data on both positive and negative self-rated health and mental health and health indicators. These capture distinct dimensions of well-being that are not adequately reflected by a single indicator. All indicators used in this study are defined and described in previous HIRI publications [8]. Since CCHS data coverage on asthma, COPD, and second-hand smoke exposure ended in 2020, only sensitivity analyses for the 2015 redesign were performed. These 20 indicators were chosen because 1) they were already part of HIRI’s indicator framework, 2) their data were available consistently across CCHS cycles over a long time period, and 3) the questionnaire items for these indicators remained sufficiently consistent across that period.

### Analysis

We began by calculating crude prevalence among adults for each of the 20 indicators across the available CCHS cycles. To allow the most direct crude comparison of prevalence estimates from the CCHS datasets collected immediately before and after the survey redesigns, neither age-standardization nor model-based age-adjustment was performed. Previous CCHS analyses before and after the 2015 redesign produced similar results when using age-standardized and crude rates [9].

Next, we estimated the absolute change in prevalence (in percentage points, PP) for the periods immediately before and after the redesigns (2013–2014 vs. 2015–2016, 2019–2021 vs. 2022). The 2019–2021 (3-year) period was used in lieu of the 2-year 2019–2020 period, to ensure coverage immediately before the 2022 redesign took place (i.e., to include data from 2021), as well as coverage of respondents from the three Territories (which are not represented in the 2021 cycle, but are represented in the 2019–2020 combined years and the 2022 cycle). Because we constructed the indicators as binary variables, we generated PP-change estimates by fitting generalized linear models (GLM) with a logit link for each indicator, regressing indicator prevalence on the CCHS cycle (e.g. 2013–2014 vs. 2015–2016, 2019–2021 vs. 2022). We estimated predicted probabilities on the risk scale using R’s *margins* command [10] to obtain the PP-change, its 95% confidence interval (CI) and p-value. We then divided the PP-change estimates by the pre-redesign prevalence estimate (from 2013–2014 and 2019–2021, respectively) and multiplied by 100 to obtain a measure of the percent (%) change.

Finally, to put the immediate changes in prevalence following the 2015 and 2022 CCHS redesigns into context with longer-term trends, we modelled the predicted prevalence for 2015–2016 and 2022 cycles using pre-redesign trends (2007–2014 and 2015–2021, respectively). We fit two GLM models with a logit link for each indicator, for each pre-redesign period. Models regressed the indicator prevalence on a discrete indexed measure of time (e.g., 2007–2008 was represented by a value of 1, 2009–2010 by a value of 2, etc.). We then used these models’ predicted probabilities to estimate the extrapolated model-projected prevalence and its 95% CI for each indicator for 2015–2016 and 2022 respectively, using R's *predict* command [11].

All analyses were performed using CCHS sampling and bootstrap weights (500 for cycles before 2015–2016, and 1000 for cycles as of 2015–2016 onwards), in R Studio (version 4.4.3) [12]. Models and R code are described in greater detail in Supplemental eTable 5.

### Decision-tree

**Fig. 1:**
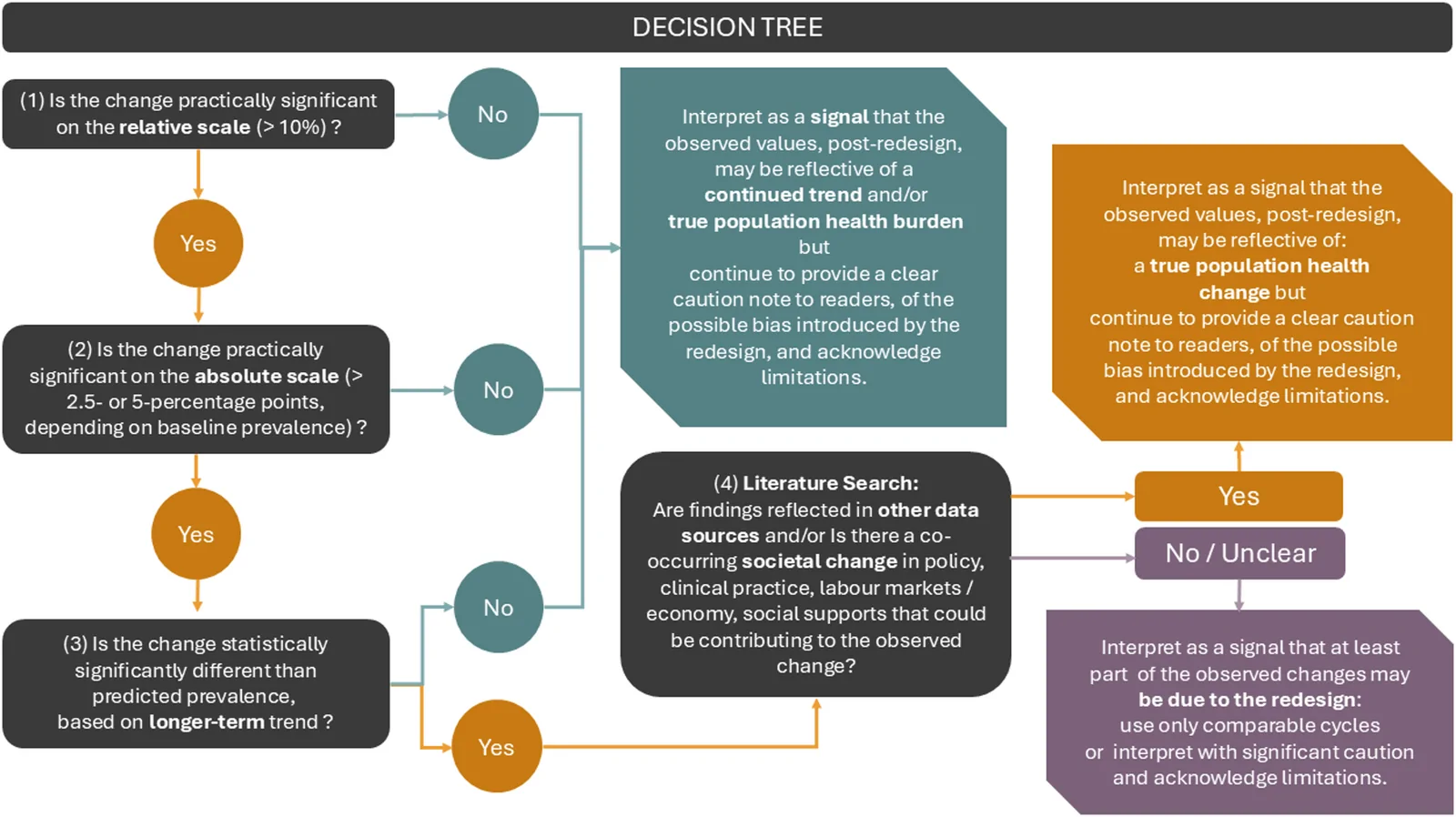
Decision-tree following sensitivity analyses of survey redesigns

When the analysis was complete, we applied a decision-tree (Fig. 1) that captured whether the change in prevalence post-redesign was practically significant on the absolute and relative scale, and whether the observed prevalence showed a statistically significant difference than what would have been expected, given pre-redesign temporal trends.

We conducted a rapid targeted review of methodological and applied epidemiologic studies on PubMed and Google Scholar platforms to identify previously used thresholds for defining meaningful absolute and relative changes in prevalence.

On the absolute scale, evidence was limited. A US-CDC-led study examining the equivalence of indicator prevalence derived from surveys compared to electronic health records, used a “margin of equivalence” threshold of a 5-percentage point (PP), with lower and upper sensitivity thresholds of 2.5- and 7.5-PP, respectively [5]. The authors recommended that the lower absolute scale threshold of 2.5-PP was likely most relevant for indicators with prevalence of less than 10% [5]. Based on this guidance, we applied a threshold of 2.5-percentage point change for indicators with a population prevalence of less than 10% in the pre-redesign period, and a threshold of 5-PP change for indicators with a population prevalence of more than 10% in the pre-redesign period. For context, in the 41 million-sized Canadian population, 2.5-PP represents just over 1 million individuals affected, and 5-PP represents approximately 2.1 million individuals affected [13].

These absolute change thresholds are imperfect. For some indicators, namely those with significant burden on quality of life, even 10,000 individuals affected (i.e., 0.2% of the Canadian population) could be considered sufficient to flag a meaningful change for public health policy. We have chosen to use this generalist, evidence-based approach, with a strong caveat that future research may warrant exploration of alternative thresholds which may vary across health or social conditions. The proposed approach is likely most appropriate in the context of health inequality analyses, which involve monitoring of a wide range of health and social indicators with varying population prevalence, and which typically consider larger absolute changes (e.g., greater than 2.5- to 5-PP) as more meaningful for health equity policy concern or intervention [14–16].

On the relative scale, some clinical studies have defined a 20% change as a meaningful difference [17], while others used a 10% change threshold [18]. In population health settings, studies have identified a 20% change as “dramatic”, a 10% change as “less dramatic”, and a change under 5% as “modest” [6]. In its Healthy People 2030 reporting, the US-CDC described a change of up to 10% on the relative scale as “being of little or no detectable change” [4]. Another CDC study used a meaningfulness threshold of 15% [5]. Considering this range of thresholds, and to avoid missing any relevant relative changes, we applied the more conservative cut-off of 10-percent (%) change. This threshold is also aligned with previous health inequality monitoring findings [19].

Thresholds on the absolute and relative scales each have their limitations. Percent change estimates are sensitive to the underlying burden of each indicator, while absolute percentage-point differences are insensitive to the underlying burden of each indicator and therefore must be interpreted with caution if a single threshold is used [5]. Following recommended health inequality monitoring practice [19], we therefore use practical significance thresholds on both scales (absolute and relative scales, respectively) to account for these limitations.

The decision-tree flagged when there was no overlap in the 95% CIs between the observed and predicted post-redesign prevalence (in 2015–2016 and 2022, respectively). Overlapping CIs were interpreted as a signal that the observed change may be aligned with the longer-term trend.

The decision-tree proposes that if there were 1) a practically significant change on the absolute and 2) relative scales (regardless of their statistical significance), and if 3) the prevalence was statistically significantly different than the predicted prevalence, a final qualitative step (4) would be needed. This step involved a targeted review of peer-reviewed literature, government reports, and administrative or surveillance data sources to assess whether similar changes were documented, and to identify potential co-occurring population-level shocks or societal dynamics that could be contributing to the observed change. These could include changes in public policy, clinical diagnostic practice, labour markets or the economy, social support or benefit regimes, as well as non-human or environmental shocks such as infectious or communicable disease outbreaks, extreme weather events or natural disasters, or longer-term climate-related risks, etc.

In our Canadian context, we conducted a scan of potential Canada-focused peer-reviewed studies on PubMed and Google Scholar platforms. We also searched Google databases and Government of Canada publication sites for Canadian reports and data briefings (Health Canada, Public Health Agency of Canada, Statistics Canada). Although many shocks and world events were occurring during the period of study [20–22], we focused specifically on Canadian evidence that could explain meaningful changes immediately post-redesigns.

If no corroborating evidence could be found through a targeted literature review, we would interpret the finding as a signal that at least a component of the observed changes may be due to the redesign. In such cases, one could choose to use only comparable cycles for analysis (i.e., not combine data pre- and post-redesign for trend monitoring). Alternatively, one could choose to use data from all available cycles, but interpret analysis of any long-term data that crossed the redesign period with significant caution, and provide clear notes to acknowledge the limitation that observed trends may be influenced by the redesign. All other instances would be interpreted as a signal that the observed values, post-redesign, may be reflective of a continued trend and/or true population health burden (or change therein). Data pre- and post-redesign could continue to be compared in reporting, while providing a clear caution note to readers of the possible bias introduced by the redesign, and acknowledging data limitations.

### Sensitivity analyses

In addition to the literature scans performed in step 4 of the decision-tree, we applied two sensitivity analyses, results of which are summarized in the Results section.

First, for indicators that crossed all three thresholds for the 2022 redesign, in particular, we also replicated a micro-trend analysis performed previously by Statistics Canada [23], wherein prevalence was estimated for: 2020, 2021, 2022 (using pre-redesign data from the CCHS 2021, collected between January and February 2022), 2022 (using data from the post-redesign 2022 cycle, collected between February and December 2022), 2023 and 2024. This granular analysis provided additional information on the direction of trends in 2022, pre- and post-redesign, that may have been missed when grouping the 2019–2021 period. Results are summarized below.

Second, we also explored if these indicators would have crossed a less conservative relative threshold of 20-percent (%) change, which has been defined as a cut-off of “minimally important change” in contrast to the 10% threshold’s “minimal detectable change” [4].

## Results

Results from the three main components of the decision-tree (percent (%) change, percentage point (PP) change and statistical significance between predicted and observed pre-redesign prevalence) are summarized in Tables 1 and 2, for the 2015 and 2022 redesigns, respectively.

**Table 1:** Pre-2015 crude prevalence, predicted and observed 2015–2016 prevalence, 2013–2014 vs. 2015–2016 prevalence change

Indicator	2007–2008	2009–2010	2011–2012	2013–2014	2015–2016	2015–2016 predicted prevalence (modeled, based on 2007–2014 data)	PP-change (95% CI) (2013–2014 vs. 2015–2016) [> 2.5- or 5-PP]	% change (2015–16 vs. 2013–14) [> 10%]	Outside of predicted 95% CI? (Yes/No)
Anxiety disorder	5.71 (5.49, 5.93)	5.31 (5.08, 5.54)	6.56 (6.28, 6.85)	7.05 (6.78, 7.33)	8.21 (7.92, 8.51)	*7.60 (7.24, 7.98)*	1.16 (0.75, 1.56) **	**16.45**	No
Asthma	7.89 (7.64, 8.14)	8.07 (7.77, 8.37)	8.14 (7.86, 8.43)	7.92 (7.65, 8.20)	8.39 (8.07, 8.71)	*8.04 (7.71, 8.39)*	0.47 (0.05, 0.88) **	5.93	No
Arthritis	16.74 (16.43, 17.04)	16.69 (16.35, 17.02)	17.07 (16.72, 17.43)	16.97 (16.63, 17.31)	21.27 (20.90, 21.65)	*17.14 (16.73, 17.56)*	4.27 (3.79, 4.76) **	**25.16**	**Yes**
Community belonging (strong/very strong)	63.37 (62.85, 63.90)	64.10 (63.55, 64.64)	64.23 (63.67, 64.80)	65.05 (64.51, 65.59)	67.11 (66.57, 67.64)	*65.47 (64.79, 66.15)*	2.06 (1.30, 2.82)**	3.17	No
COPD	*NA*	4.24 (4.04, 4.44)	4.22 (4,00 4.45)	4.13 (3.92, 4.34)	4.14 (3.92, 4.35)	*4.09 (3.79, 4.40)*	0.01 (−0.29, 0.30)**	0.24	No
Diabetes (excluding gestational diabetes)	6.37 (6.16, 6.58)	6.62 (6.38, 6.86)	6.86 (6.60, 7.13)	7.09 (6.84, 7.34)	7.31 (7.06, 7.57)	*7.36 (7.05, 7.68)*	0.22 (−0.13, 0.58)	3.10	No
Diabetes (including gestational diabetes)	6.46 (6.24, 6.68)	6.68 (6.44, 6.93)	6.94 (6.67, 7.20)	7.18 (6.93, 7.43)	7.41 (7.15, 7.67)	*7.44 (7.12, 7.76)*	0.23 (−0.13, 0.60)	3.20	No
Food insecurity (moderate/severe)	6.92 (6.60, 7.23)	6.90 (6.60, 7.20)	7.56 (7.23, 7.89)	7.27 (6.91, 7.62)	7.54 (7.18, 7.90)	*7.62 (7.18, 8.09)*	0.27 (−0.23, 0.70)	3.71	No
Heart disease	5.28 (5.09, 5.46)	5.21 (5.01, 5.40)	5.34 (5.13, 5.56)	5.21 (5.01, 5.41)	4.94 (4.74, 5.14)	*5.24 (5.00, 5.50)*	−0.27 (−0.55, 0.00)	−5.18	No
Hypertension	18.88 (18.53, 19.24)	19.51 (19.12, 19.89)	20.10 (19.75, 20.46)	20.30 (19.95, 20.65)	21.20 (20.84, 21.56)	*20.92 (20.47, 21.39)*	0.90 (0.40, 1.40) **	4.43	No
Life satisfaction (satisfied/very satisfied)	*NA*	91.67 (91.37, 91.98)	91.99 (91.67, 92.30)	91.64 (91.31, 91.98)	92.49 (92.21, 92.77)	*91.73 (91.20, 92.23)*	0.85 (0.41, 1.30) **	0.93	No
Life stress (quite/very stressful)	22.82 (22.37, 23.28)	23.82 (23.36, 24.27)	23.46 (22.98, 23.93)	23.17 (22.66, 23.67)	22.18 (21.72, 22.64)	*23.47 (22.87, 24.07)*	−0.99 (−1.66, −0.31) **	−4.27	**Yes T**
Mood disorder	7.05 (6.82, 7.28)	6.98 (6.71, 7.25)	7.56 (7.29, 7.83)	8.10 (7.81, 8.40)	8.48 (8.19, 8.77)	*8.41 (8.04, 8.80)*	0.37 (−0.02, 0.77)	4.57	No
Obesity	23.23 (22.84, 23.62)	23.84 (23.38, 24.30)	24.79 (24.32, 25.26)	26.14 (25.65, 26.63)	26.45 (25.98, 26.93)	*26.99 (26.40, 27.59)*	0.31 (−0.37, 0.99)	1.19	No
Second-hand smoke exposure (home)	5.76 (5.51, 6.01)	5.10 (4.83, 5.37)	4.37 (4.10, 4.64)	3.63 (3.41, 3.84)	3.12 (2.90, 3.34)	*3.17 (2.96, 3.39)*	−0.51 (−0.89, −0.20)**	**−14.05**	No
Self-rated health (fair/poor)	12.13 (11.83, 12.42)	12.03 (11.71, 12.35)	11.94 (11.57, 12.30)	11.89 (11.57, 12.21)	11.58 (11.25, 11.92)	*11.80 (11.41, 12.19)*	−0.31 (−0.76, 0.15)	−2.61	No
Self-rated health (very good/excellent)	58.29 (57.79, 58.79)	59.40 (58.89, 59.91)	59.00 (58.42, 59.57)	58.40 (57.89, 58.91)	60.49 (59.97, 61.02)	*58.73 (58.09, 59.38)*	2.09 (1.38, 2.81)**	3.58	**Yes T**
Self-rated mental health (fair/poor)	5.08 (4.86, 5.30)	5.43 (5.19, 5.67)	5.91 (5.66, 6.17)	6.42 (6.15, 6.69)	6.60 (6.32, 6.88)	*6.93 (6.59, 7.28)*	0.17 (−0.21, 0.56)	2.65	No
Self-rated mental health (very good/excellent)	74.14 (73.70, 74.59)	73.57 (73.09, 74.06)	71.68 (71.13, 72.24)	70.76 (70.24, 71.28)	71.15 (70.64, 71.66)	*69.45 (68.77, 70.11)*	0.39 (−0.29, 1.08)	0.55	No
Smoking (daily or occasionally in past 30 days)	21.88 (21.45, 22.30)	20.47 (20.05, 20.89)	20.02 (19.56, 20.49)	18.49 (18.04, 18.95)	17.13 (16.71, 17.54)	*17.67 (17.15, 18.20)*	−1.37 (2.00, −0.74)**	−7.41	No

% refers to percent; "PP" refers to percentage point; CI refers to confidence interval; ** Indicates PP-change values that are statistically significant (p < 0.05, i.e., alpha = 0.05); "T" indicates values that fall very close to the threshold in question, e.g. ± 0.5-PP (interpret with caution); Bolded in-table text indicates when a decision-matrix threshold was crossed; > 10% refers to the relative scale threshold of 10% change; absolute scale thresholds were > 5-percentage point (PP) change for indicators with > 10% prevalence in 2013–2014, and > 2.5-PP change for indicators with < 10% prevalence in 2013–2014; NA indicates where the data for an indicator was not available.

**Table 2:** Pre-2022 crude prevalence, predicted and observed 2022 prevalence, 2019–2021 vs. 2022 prevalence change

Indicator	2015–2016	2017–2018	2019–2021	2022	2022 predicted prevalence (modeled, based on 2015–2019 data)	PP-change (95% CI) (2019–2021 vs. 2022) [>2.5- or 5-PP]	% change (2019–2021 vs. 2022) [>10%]	Outside of predicted 95% CI? (Yes/No)
Anxiety disorder	8.21 (7.92, 8.51)	9.09 (8.80, 9.37)	10.06 (9.73, 10.39) T	15.08 (14.56, 15.60)	*11.11 (10.58, 11.67)*	4.65 (4.15, 5.15)**	**43.87**	**Yes**
Arthritis	21.27 (20.90, 21.65)	19.75 (19.37, 20.13)	20.28 (19.96, 20.60)	20.57 (20.08, 21.07)	*19.48 (19.00, 19.97)*	0.29 (−0.29, 0.87)	1.43	**Yes T**
Community belonging (strong/very strong)	67.11 (66.57, 67.64)	67.59 (67.08, 68.10)	68.56 (68.05, 69.06)	63.44 (62.68, 64.20)	*69.19 (68.40, 69.98)*	−5.03 (−5.90, −4.16)**	−7.39	**Yes**
Diabetes (excluding gestational diabetes)	7.31 (7.06, 7.57)	7.57 (7.31, 7.82)	7.83 (7.61, 8.06)	8.24 (7.88, 8.61)	*8.11 (7.74, 8.49)*	0.41 (−0.02, 0.83)	5.24	No
Diabetes (including gestational diabetes)	7.41 (7.15, 7.67)	7.71 (7.45, 7.97)	7.96 (7.73, 8.19)	8.36 (7.99, 8.72)	*8.26 (7.89, 8.64)*	0.39 (−0.04, 0.82)	4.90	No
Food insecurity (moderate/severe)	7.54 (7.18, 7.90)	8.07 (7.74, 8.40)	6.20 (5.93, 6.48)	10.91 (10.41, 11.40)	*5.91 (5.54, 6.29)*	**4.27 (3.81, 4.72)**	**68.87**	**Yes**
Heart disease	4.94 (4.74, 5.14)	4.89 (4.70, 5.08)	5.12 (4.95, 5.29)	5.40 (5.13, 5.68)	*5.17 (4.90, 5.45)*	0.28 (−0.04, 0.60)	5.47	No
Hypertension	21.20 (20.84, 21.56)	21.61 (21.25, 21.97)	21.68 (21.36, 22.01)	22.57 (22.08, 23.06)	*21.98 (21.47, 22.49)*	0.88 (0.29, 1.48)**	4.06	No
Life satisfaction (satisfied/very satisfied)	92.49 (92.21, 92.77)	92.77 (92.48, 93.05)	92.73 (92.47, 92.99)	87.28 (86.76, 87.80)	*92.90 (92.50, 93.29)*	−4.92 (−5.39, −4.46)**	−5.31	**Yes**
Life stress (quite/very stressful)	22.18 (21.72, 22.64)	21.99 (21.54, 22.44)	21.26 (20.84, 21.67)	22.25 (21.62, 22.87)	*20.89 (20.25, 21.55)*	0.98 (0.25, 1.72)**	4.61	**Yes T**
Mood disorder	8.48 (8.19, 8.77)	9.17 (8.84, 9.51)	9.72 (9.39, 10.04) T	12.02 (11.54, 12.51)	*10.43 (9.92, 10.95)*	2.21 (1.70, 2.72)**	**22.76**	**Yes**
Obesity	26.45 (25.98, 26.93)	26.95 (26.45, 27.46)	28.64 (28.17, 29.11)	30.32 (29.63, 31.02)	*29.59 (28.86, 30.33)*	1.67 (0.84, 2.51)**	5.83	No
Self-rated health (fair/poor)	11.58 (11.25, 11.92)	11.71 (11.39, 12.03)	11.52 (11.23, 11.81)	14.56 (14.04, 15.08)	*11.54 (11.10, 11.99)*	2.91 (2.35, 3.46)**	**25.26**	**Yes**
Self-rated health (very good/excellent)	60.49 (59.97, 61.02)	59.81 (59.28, 60.35)	60.03 (59.54, 60.51)	53.74 (52.96, 54.51)	*59.65 (58.89, 60.41)*	−6.22 (−7.12, −5.32)**	**−10.36 T**	**Yes**
Self-rated mental health (fair/poor)	6.60 (6.32, 6.88)	7.49 (7.19, 7.79)	9.91 (9.58, 10.25) T	14.45 (13.89, 15.02)	*11.91 (11.31, 12.53)*	4.23 (3.68, 4.79)**	**42.68**	**Yes**
Self-rated mental health (very good/excellent)	71.15 (70.64, 71.66)	69.00 (68.51, 69.49)	63.49 (62.97, 64.00)	54.61 (53.81, 55.42)	*59.82 (59.00, 60.64)*	−8.69 (−9.60, −7.79)**	**−13.69**	**Yes**
Smoking (daily or occasionally in past 30 days)	17.13 (16.71, 17.54)	15.78 (15.36, 16.20)	12.81 (12.47, 13.16)	12.47 (11.98, 12.95)	11.32 (10.87, 11.78)	−0.35 (−0.93, 0.23)	−2.73	**Yes T**

% refers to percent; "PP" refers to percentage point; CI refers to confidence interval; ** Indicates PP-change values that are statistically significant (*p* < 0.05); "T" indicates values that fall very close to the threshold in question, e.g. ± 0.5-PP (interpret with caution); Bolded in-table text indicates when a decision-matrix threshold was crossed; > 10% refers to the relative scale threshold of 10% change; absolute scale thresholds were > 5-percentage point (PP) change for indicators with > 10% prevalence in 2019–2021, and > 2.5-PP change for indicators with < 10% prevalence in 2019–2021. Indicators of asthma, COPD and second-hand smoke exposure in the home were excluded from this table, as their data coverage in the CCHS ended in 2020.

For the 2015 redesign, no indicators met all three criteria (Table 1). There was a 25% increase in observed arthritis prevalence between 2013–2014 and 2015–2016, which was greater than what was predicted given 2007–2014 trends (21.3% instead of the predicted 17.1%). However, the absolute change fell below the 5-PP threshold. Anxiety, life stress, second-hand smoke exposure in the home, and very good or excellent self-rated health met either the %-change or statistical significance predicted difference thresholds, only.

For the 2022 redesign, self-rated mental health (very good or excellent) and (fair/poor), self-rated health (very good/excellent), and food insecurity (moderate or severe), met all three criteria (Table 2). Regarding self-rated mental health, although there has been a downward trend since 2007–2008 (from 74.1% in 2007–2008 to 63.5% in 2019–2021) (Tables 1 and 2), 2022 was associated with a larger decrease in prevalence than predicted (from 63.5% prevalence in 2019–2021 to 53.7% in 2022, instead of the predicted 59.7%) (Table 2). CCHS data from the provinces only, suggest it stabilized afterwards (53.4% in 2023, 53.7% in 2024) (Supplemental eTable 6). Micro-trend sensitivity analyses suggest that there was a decline between 2020 and 2022, before the redesign (64.9% in 2020, 59.2% in 2021, 56.3% in 2022 pre-redesign, 54.6% in 2022 post-redesign) (Supplemental eTable 7). The relative change value of −13.7% (Table 2) would not have crossed the “minimally important change” threshold of 20%.

Secondly, for fair/poor self-rated mental health, a larger increase was observed than expected post-redesign (from 9.9% in 2019–2021 to 14.5% in 2022, instead of the predicted 11.9% for 2022) (Table 2). That said, the pre-redesign (2019–2021) prevalence (9.9%) was on the cusp of the prevalence threshold used; if the 5-PP cut-off for greater-than-10%-prevalence indicators were used, this indicator would not have met all three criteria.

Micro-trend sensitivity analyses do not indicate a clear increase between 2020 and 2022, before the redesign (9.8% in 2020, 11.7% in 2021, 11.7% in 2022, pre-redesign, 14.5% in 2022, post-redesign) (Supplemental eTable 4). The relative change value of –42.7% pre-post 2022 (Table 2) crosses the “minimally important change” threshold of 20%.

Third, self-rated health (very good/excellent) prevalence was largely stable between 2015–2016 (60.5%) and 2019–2021 (60%). The observed decrease from 60% in 2019–2021 to 53.7% in 2022, a 10.4% decrease, was significantly larger than what was predicted for 2022 (Table 2). Here too, the relative change was on the 10%-threshold cusp and would not have met the “minimally important change” threshold of 20%. Micro-trend sensitivity analyses do not indicate a clear decrease between 2020 and 2022, pre-redesign (Supplemental eTable 4).

Finally, there was a small but steady increase in moderate to severe food insecurity between 2007–2008 and 2017–2018 (from 6.9% to 8.1%), followed by a dip in 2019–2021 to below pre-2007–2008 levels (6.2%) (Tables 1 and 2). Using data from 2015 to 2021, the predicted prevalence for 2022 was 5.9%, which was significantly lower than the observed food insecurity prevalence of 10.9% in 2022. Of note is the continued increase in food insecurity in post-pandemic years (12.6% in 2023, 13.8% in 2024) (Supplemental eTable 5). The 68.9% change between 2019–2021 and 2022 crosses the “minimally important change” threshold of 20%. Micro-trend sensitivity analyses suggest that there was an increase in 2022, before the redesign (6.0% in 2020, 5.7% in 2021, 6.0% in 2022 pre-redesign, 10.9% in 2022 post-redesign) (Supplemental eTable 7).

## Discussion

This study explores the use of sensitivity analyses, combined with a decision-tree framework that balanced considerations of both statistical and practical significance, to assess the comparability of CCHS survey cycles before and after major redesigns in 2015 and 2022. Its straightforward design, with corresponding statistical analyses and literature scans, provides an efficient, structured, and reproducible approach to assessing the potential impacts of redesigns on observed trends across multiple indicators. When examining pre- and post-change differences following the 2015 redesign, none of the 20 selected indicators met all three of the decision-tree criteria. In contrast, following the 2022 redesign, food insecurity, self-rated mental health, and self-rated health met all three decision-tree criteria.

Regarding the observed increase in food insecurity in 2022, Statistics Canada had previously reported on this finding [24]. Using Consumer Price Index (CPI) data, they found that the spike in food insecurity in 2022 mirrored the increase in all-items pricing, as inflation rose to approximately 6% to 7% for all-items, and to 8% to 11% for food purchased in stores. Inflation stayed high for food prices in 2023 (between 11.4% in January to 4.7% in December) [25], which may help explain the continued high prevalence of food insecurity that year (13%) (Supplemental eTable 6). Although the effects of the change in survey mode may be at play for this indicator, literature on survey mode effects on food insecurity measurement is limited, and existing studies suggest impacts may be small [26]. These findings indicate that the observed increase in food insecurity for Canada overall, as of 2022, may be due in part to rising food price inflation, but that the residual effects of the redesign cannot be ruled out. Supplemental eTable 8 provides an example of a caution note that can be used in this scenario. Regarding the observed change in self-rated mental health observed post-2022 redesign, findings from other data sources against which to compare these findings are mixed. On one hand, this finding aligns with those from past research using data from 2019 to 2022, before the CCHS redesign [23]. Specifically, prevalence of excellent or very good mental health among those aged 12 and above, declined statistically significantly from 67% in January to December 2019, to 64% in September to December 2020, and to 58.1% in September 2021 to February 2022, when COVID-19 cases and hospitalizations surged and public health restrictions increased [23]. This could signal that the 54% prevalence observed for adults in 2022 may be following a similar trend, related to the COVID-19 pandemic, and not simply because of the change in survey design or mode (i.e., there is a 4.1-PP gap between 58.1% and 54.0%). Further, a Statistics Canada study of the consumer price index during this period noted a peak in inflation in 2022, for food, shelter and transportation, and many reported worries in meeting day-to-day expenses, which could have contributed to a decline in self-rated mental health [24]. Canadian polling supports the finding that new pressures (such as inflation and food insecurity) emerged as dominant stressors in 2022 [27, 28].

In contrast, quarterly data from the Canadian Social Survey (CSS), which was launched in 2021, with respondents aged 15 years and above, from provinces only, do not demonstrate such a clear or consistent decrease before and after 2022 [29]. Like the CCHS as of 2022, the CSS uses an electronic questionnaire (EQ) with telephone interview follow-up for non-response. Unfortunately, the CSS was launched in 2021, and longer-term trends using this data source are not available for comparison. It is also a general social survey rather than a health survey, which may affect how respondents answer questions on mental health [24]. That said, research on mode effects on self-rated mental health response suggest that respondents may indeed be more likely to report better mental health when responding to in-person or telephone interviews compared to EQs, due to social desirability bias and stigma [30–34]. In sum, although the observed change likely reflects a continued decline in self-rated mental health in Canada, the estimates and the magnitude of the observed changes post-2022 are likely sensitive to the change in survey mode. Caution notes acknowledging these nuances are needed for this indicator for public health reporting (an example of which is described in Supplemental eTable 8).

Lastly, regarding the observed meaningful declines in self-rated health post-2022 redesign, this finding is aligned with research pertaining to the COVID-19 pandemic period and its impacts on the physical health of Canadians. Data from the Canadian COVID-19 Antibody and Health Survey indicate that by the summer of 2022, about 1 in 3 Canadian adults (38.7%) had experienced a confirmed or suspected SARS CoV-2 infection [35]. In the fall of 2022, about 1 in 7 adults (14.8%) experienced longer-term symptoms, known as “Post COVID-19 condition” (PCC) or “long COVID” [36]. PCC is associated with limitations in daily activities [35, 36] and lower self-rated health [37]. Further, regarding the broader changes in Canadian society, pre-pandemic research observed a strong association between food insecurity and poorer self-rated health [38]. Increases in food insecurity associated with price inflation in 2022 [24] may also be contributing to the declines in self-rated health during this period.

That said, the significant drop in self-rated health in 2022, observed in the CCHS, is not visible in available data from the CSS, from the second quarter (Q2) of 2021 to Q3 of 2023 [39]. Unfortunately, data are not available for all quarters, over these years. If we focus exclusively on quarters with available CSS data across calendar years, a 6.6-PP decrease in very good or excellent self-rated health is observable between Q2 of 2021 (57.1%) and Q2 of 2022 (50.5%) (similar to the observed 6.2-PP decrease between 2019–2021 and 2022 in the CCHS), but for other quarters the change is smaller (e.g., 2.4-PP decrease between Q4s of 2021 and 2022) or reversed (e.g., 2.4-PP increase between Q3s of 2021 and 2022). In sum, although it may be plausible that self-rated health declined before and after 2022, the estimates and the magnitude of the observed changes post-2022 are likely sensitive to the redesign and change in survey mode. Caution notes acknowledging these nuances are needed for this indicator for public health reporting (example provided in Supplemental eTable 8).

### Limitations

This analysis has several limitations that should be considered when interpreting the results and their applicability to CCHS estimates across survey redesigns. First, the decision-tree framework was developed specifically in the context of HIRI’s routine national health inequality surveillance activities, for indicators that are monitored using the CCHS. It is intended to inform monitoring of population-level trends rather than to support causal inference or quasi-experimental policy evaluation, and it may not be directly transferable to other surveys or redesign contexts. Analysts conducting routine public health surveillance analyses using other datasets that have undergone redesigns, should consider the types of design changes introduced and the timing of those changes. For example, changes in the characteristics of the survey’s target population (e.g., shifts in age restrictions) may require that the decision-tree analyses be replicated across various sample strata (e.g., defined by age groups). Researchers may also want to explore whether the thresholds used in this study should be adapted to their local context or indicators of interest (e.g., using more conservative absolute change thresholds than 2.5- or 5-PP).

Second, the analyses and decision-tree are best-suited for identifying a departure from what we would have expected, given pre-redesign trends or prevalence values. For example, if the pre-redesign trends predict that the 2022 prevalence should be 8%, and we indeed observed an 8% prevalence for that indicator, our decision-tree framework would not flag this as a meaningful change. However, this framework could miss situations in which a true population change occurred at the same moment as the redesign (e.g., prevalence rose to 15% instead of the predicted 8%), but that the survey redesign—due to a change in survey mode, for example—could have led to an undercounting of cases, leading to the observed post-redesign prevalence estimate of 8%. It is therefore advised to include a note that trend analyses should be interpreted with caution, and with consideration of relevant literature and subject-matter expertise, when the analyses include cycles before and after CCHS redesigns.

Third, our analyses did not explicitly account for the decline in survey response and share rates (i.e., the proportion of a total that is accounted for by a subgroup or category) observed in more recent CCHS cycles. Declining response rates can introduce bias whereby the burden of inequalities is overestimated or underestimated [40]. Statistics Canada has addressed these challenges through collection redesigns, including increased use of electronic questionnaires, and by placing greater emphasis on achieving representativeness of the target population through weighting adjustments [40, 41]. While lower response rates do not necessarily imply biased estimates, and higher response rates do not guarantee the absence of bias, residual nonresponse bias remains a consideration.

Fourth, the 2019–2021 period was used to ensure data coverage of the Territories and of data immediately before the 2022 redesign. However, pooling prevalence estimates across this period may have masked granular year-to-year variation, potentially affecting the estimated magnitude of the change pre- vs. post-2022. The micro-trend analyses of 2020 to 2024 data, separately, helped mitigate this issue, and provided additional information on whether a more gradual change was occurring during this period.

Next, the third criterion of the decision-tree involved predictive modeling. Indicators with relatively stable prevalence over time (i.e., with small peaks or valleys), such as arthritis or life-stress, did not have strong convergence of predicted versus observed estimates. The modeling-based component of the decision may have been most effective in identifying meaningful changes when there was a clear downward or upward trend in prevalence in the pre-redesign period. This limitation is mitigated by considering the modeling output in conjunction with the results of the other two analyses. Future research and adaptations of the decision-tree, particularly those for more technical audiences, could incorporate additional information from the pre-redesign models, such as variability of the pre-redesign estimates around the trend, when interpreting differences between observed and predicted estimates for the post-redesign period.

In addition, the analyses were conducted exclusively among adults aged 18 years and older. It is therefore unclear whether these findings are generalizable to other stages of the life course, such as youth or older adults, or to other distinct groups. Further, for indicators with uneven provincial coverage in certain cycles (e.g. anxiety in 2023, food insecurity in 2013–2020, and second-hand smoke exposure in 2017–2020), period-specific estimates should be interpreted with caution, as their generalizability to the Canadian population overall may be limited.

Finally, as mentioned previously, the interpretation of what constitutes a “meaningful” change threshold may depend on the context and aims of the study. The thresholds used in this study are not intended to be definitive guidelines, but rather evidence-based, reproducible examples that others may adapt or build on. In any case, researchers should be explicit about their reasoning for setting specific “meaningful change” thresholds. Further, as the literature scan component of the decision-tree can yield mixed results, requiring interpretation and judgement, the proposed decision-tree remains a useful decision-support tool rather than a prescriptive set of rules. The decision-tree does not eliminate the need for subject-matter expertise and contextual interpretation when determining whether CCHS cycles should be combined, and how caution notes should be framed.

## Conclusion

This study demonstrates how sensitivity analyses combined with a decision-tree framework can support decisions concerning combining and interpreting long-term data when major methodological changes in data sources occur. Using the CCHS as a case study, we demonstrate how changes can be measured on relative and absolute scales, and with consideration of pre-redesign trends that may signal plausible underlying population change. Our findings suggest that the 2015 redesign did not produce practically meaningful changes for the indicators examined. The 2022 redesign warrants closer scrutiny and interpretation for a small subset of outcomes, notably food insecurity and self-rated physical and mental health.

Overall, the proposed framework offers a pragmatic approach for long-term health inequality monitoring amidst evolving data sources. While not eliminating uncertainty, it provides a reproducible way to document and communicate caution to users. Continued refinement of the framework will be valuable to adapt this approach to other data sources or indicators, to support rigorous, transparent and policy-relevant analyses over time.

## Supplementary Information

Supplementary Material 1.

## Data Availability

The data supporting the conclusions of this article are from the Canadian Community Health Survey (CCHS), conducted by Statistics Canada. Access to the full CCHS dataset is restricted under Canada’s Statistics Act and is available only to approved researchers. Researchers must obtain project approval, security clearance, and be sworn in as deemed employees of Statistics Canada before accessing the data. These data cannot be publicly shared by the authors. HIRI's Health Inequalities Data Tool (HIDT) contains data on indicators of health status and health determinants. Data are disaggregated by a range of social and economic characteristics (i.e., social stratifiers) meaningful to health equity. Data are available here: Health Inequalities Data Tool (https://health-infobase.canada.ca/health-inequalities/data-tool/).

## Abbreviations

CCHS: Canadian Community Health Survey
CDC: U.S. Centres for Disease Control and Prevention
CI: Confidence interval
COPD: Chronic obstructive pulmonary disorder
CPI: Canadian Price Index
CSS: Canadian Social Survey
GLM: Generalized linear model
HIDT: Health Inequalities Data Tool
HIRI: Health Inequalities Reporting Initiative
PHAC: Public Health Agency of Canada
PP: Percentage point
