# Supplementary material for: A decision-tree for trend analyses following major survey redesigns: an analysis using the Canadian Community Health Survey (CCHS)
Source: BMC Public Health. 2026 Aug 8;26:3205. doi: 10.1186/s12889-026-28851-7 (PMC13733081; doi:10.1186/s12889-026-28851-7)
Supplement: Supplementary file 1 [file 12889_2026_28851_MOESM1_ESM.docx]

## Supplementary Materials

**Contents:**

**eTable 1:** Summary of key changes to the CCHS survey in 2015 and 2022

**eTable 2:** A non-exhaustive snapshot of peer-reviewed studies, reports, and data tools or dashboard use of CCHS cycles before and after 2015 and/or 2022 redesigns

**eTable 3:** A non-exhaustive snapshot of approved Statistics Canada Research Data Centre projects using CCHS cycles before and after the 2015 and/or 2022 redesigns

**eTable 4:** Indicator provincial and territorial data coverage

**eTable 5:** Regression models used for each of the decision-tree criteria, and corresponding R code

**eTable 6:** Crude prevalence of all indicators, across all cycles, from 2007-2008 to 2024 among adults, aged 18 years and older)

**eTable 7:** Micro-trend sensitivity analyses of crude prevalence of select indicators, in 2020-2021, 2022 (pre-redesign), 2022 (post-redesign), 2023-2024 among adults.

**eTable 8:** Examples of proposed caution notes for HIRI data users, for indicators that met or did not meet all decision-free flags.

**eTable 1:** Summary of key changes to the CCHS survey in 2015 and 2022

**2015: Sampling, timing, content, target population revisions**

**Sampling**

- Before 2015, the CCHS used three sampling frames to select households for data collection (an area frame, a list frame of telephone numbers, and a random digit dialling sampling frame). Once households were identified, a person (12 and older) was selected for interview, according to age-based selection probabilities.
- From 2015 onwards, the CCHS sample is selected using two different frames: an area frame, for adults (18+), and the Canadian Child Tax Benefit (CCTB) frame, for those younger than 18 years.
  - Using the area frame, a sample of dwellings is selected to target the population aged 18 and over. During collection, all members of the dwelling are listed and a person aged 18 years or over is automatically selected using various selection probabilities based on age and household composition.
  - Samples are drawn from the CCTB frame every 3 months. The CCTB files contain a list of all program beneficiaries with their names, addresses and phone numbers. The CCTB list is used to select directly the youths who will be interviewed over the phone. The CCTB frame is used to sample persons aged 12 to 17 years. One child is then pre-selected to complete the survey.
- The CCHS area frame aligns the survey with the Labour Force Survey (LFS). The LFS sampling plan is a complex two stage stratified cluster design in which each stratum is formed of clusters, and the dwelling is the final sampling unit. The LFS first selects clusters using a sampling method with a probability proportional to size (PPS). Then, dwelling lists are prepared for each cluster. The final sample is chosen using a systematic sampling of households/dwellings in the cluster.
- For CCHS, LFS clusters are grouped in each health region (HR). Then, a sample of clusters and systematic dwellings are selected in each HR. The process maximizes the overlap between the clusters selected by both surveys and ensures that the same dwelling is selected only once. As of 2015, the CCHS’s multi-stage sample allocation strategy gives relatively fair sample distribution to the health regions (HR) and the provinces. For each age group (18 and over, 12 to 17), the sample is first allocated among the provinces using a power allocation of 0.75 according to the size of their respective population; then each province's sample is allocated among its HRs using a power allocation of 0.35 according to the size of the population in each HR. For the CCTB frame, an HR is assigned to each child in the target population based on the address. The CCTB frame is then stratified by HR. A simple random sample (SRS) of children aged 12 to 17 is selected within each HR.
- With the 2015 CCHS redesign, a dynamic telephone number strategy was developed. Starting in 2015, the 18+ years old sample can have up to 2 phone numbers, while the CCTB samples can have up to 4 telephone numbers. Prior to this, only 1 phone number was associated with a given household/respondent. This increases the number of available telephone numbers CATI interviewers can call to maximize their potential ability to reach the selected households or respondents. In addition, the functionality of transferring CATI (telephone) area frame cases to CAPI field interviewers was added. For cases that were contacted in CATI and reached the wrong address, had the wrong telephone number, or could not be reached, these cases would be transferred to the field CAPI interviewers who would then physically visit the listed address to try and collect a response. This ensures that all cases will have sufficient contact attempts

**Timing**

- Prior to 2015, there were six two-month collection periods.
- Starting in 2015, survey collection was divided into four non-overlapping three-month periods.

**Non-response**

- In 2015, non-response adjustments based on separate models for telephone and personal interviews, were introduced. New data sources were also integrated to design these adjustments.

**Content**

- The CCHS underwent major content revisions to its modules in 2015 (70% of modules had some level of change). As such, the formulation and wording of certain questionnaire items were updated. Some new modules were created.
- Variable naming conventions changed in 2015.

**2022: Timing, coverage, survey mode, content revisions**

**Timing**

- Typically, CCHS data collection runs across all months of the calendar year (see details below on Collection Periods).
- In 2022, due to operational constraints, the start of collection for the 2022 CCHS was delayed until February 1st, 2022.
- In 2023 and 2024, data collection continued as in the past.

**Territory coverage**

- Unique to the 2022 among the single-year cycles is its increased sample size and coverage for the three Territories, to have full coverage of the North in 2022.

**Survey mode**

- Prior to 2022, CCHS data collection used computer-assisted telephone interview (CATI)/computer-assisted personal interview (CAPI) modes.
- As of 2022, CCHS data are collected either through an electronic questionnaire (EQ) directly online or assisted by a Statistics Canada interviewer through CATI (computer assisted telephone interviewing) or CAPI (computer assisted personal interviewing), following non-response. Note that CAPI was re-introduced in the final collection period of 2022.
- For the 2022 cycle onwards, a code that gives access to the online questionnaire is sent via letter mail to households. The letter asks that a household member access the questionnaire online and answer preliminary questions, including a listing of all people that reside in that dwelling. A household member aged 18 or older is then randomly selected to participate in the survey. An email containing a second access code is sent to the selected respondent so that they may answer their portion online. A Statistics Canada interviewer may follow up by calling, emailing, texting, or visiting the respondent if a completed online questionnaire is not received within a certain period.

**Content**

- The transition to EQ required a substantial format update to all modules. Namely:
  - Interviewer notes for probing respondents were replaced with condensed on-screen help text;
  - Questions and corresponding answer categories were shortened for clarity;
  - Repetitive questions were combined under one header rather than having a series of individual questions (e.g., using a grid to ask multiple related questions).
  - The number of edits to verify respondent’s answers was reduced;
  - Other than questions required to verify the respondent’s identity, non-response was allowed for all content (i.e., the ability to skip questions)
- The CCHS underwent major content revisions to several of its modules in 2022, including those on chronic conditions, smoking, life satisfaction, sexual orientation, etc.:
  - In 2022, the new Chronic Conditions 1 Module (CC1) was introduced.
  - In 2022 and 2023, the anxiety disorder item was included in the new Chronic Conditions 1 Module (CC1). In 2024, it returned to the Chronic Conditions (CCC) module.
  - In 2022, the CCC module was asked first, followed by the CC1 module. In previous cycles, only the CCC module was asked.

**Sampling**

- For the adult sample, the CCHS returned to using the Labour Force Survey (LFS) area frame in 2022 instead of the Dwelling Universe File which was used in 2021.
- As described above for 2015, the 2022 CCHS still uses two sampling frames for its sample selection: an area frame for the Canadian population aged 18 and over, and a frame of telephone numbers from Canada Child Benefit (CCB) records for the 12-17 population.
- Between April and June 2022, the collection period did not include persons selected from the CCB list frame (only respondents aged 18 and older were surveyed in these months).

**CCHS response rates over time**

- 2007-2008: 77.6%
- 2009-2010: 73.2%
- 2011-2012: 68.4%
- 2013-2014: 66.2%
- 2015-2016: 59.5%
- 2017: 62.8%
- 2018: 58.8%
- 2019: 54.4%
- 2020: 29.7%
- 2021: 24.1%
- 2022: 42.7%
- 2023: 40.6%
- 2024: 37.8%

For comparison, the Canadian Social Survey (EQ-based)’s response rates were as follows:

- 2021 Q2: The response rate for CSS-CW is estimated at 58.9%.
- 2021 Q3: The response rate for CSS-WAPT is estimated at 55.5%.
- 2021 Q4: The response rate for CSS-WUWFT is estimated at 58.4%.
- 2022: response rates unavailable
- 2023 Q2: The response rate for CSS-QLEU is estimated at 49.4%.
- 2023 Q3: The response rate for CSS-QLVHCT is estimated at 51.7%.
- 2023 Q4: The response rate for CSS-QLRET is estimated at 54%.

**CCHS collection periods, over time**

Up until 2020, CCHS collection periods began in January and ended in December of the collection year. No interruptions were reported in the CCHS User Guides.

COVID-19 and the Census caused interruptions in CCHS collection, reflected below, before returning to the standard periods in 2023:

- 2020: January 2020 to March 2020 and September 2020 to December 2020
- 2021: January 2021 to February 2022 (paused in May 2021 due to the Census) and June 2021 to February 2022
- 2022: February 2022 to December 2022 (missing January 2022)
- 2023: January 2023 to December 2023
- 2024: January 2024 to December 2024

A summary of changes across CCHS cycles is available here:

[Summary of changes - Surveys and statistical programs - Canadian Community Health Survey - Annual Component (CCHS)](https://www23.statcan.gc.ca/imdb/p2SV.pl?Function=getMainChange&Id=1552386) ; <https://www23.statcan.gc.ca/imdb/p2SV.pl?Function=getMainChange&Id=1552386>

**eTable 2:** A non-exhaustive snapshot of peer-reviewed studies, reports, and data tools or dashboard use of CCHS cycles before and after 2015 and/or 2022 redesigns

| **Publications** | **Combines cycles pre-2015 and 2015?** | **Combines cycles pre-2022 and 2022?** |
| --- | --- | --- |
| Public Health Agency of Canada. (2025) Obesity Statistics in Canada: Report. <https://www.canada.ca/en/public-health/services/publications/healthy-living/obesity-statistics-canada.html#a6.3> (e.g., Figure 2.8, 2000-2001 to 2019-2020) | **✅** | **✖️** |
| Public Health Agency of Canada (2025) Health of People in Canada Dashboard [Explore the dashboard: Health of people in Canada dashboard – Canada.ca](https://health-infobase.canada.ca/health-of-people-in-canada-dashboard/?topic=Health%2520Status&indicator=Perceived%2520Mental%2520Health&graph=svg-trendLine) (e.g., self-rated mental health between 2015 and 2023) | **✖️** | **✅** |
| Statistics Canada (2025) Health of Canadians: Report.  <https://www150.statcan.gc.ca/n1/pub/82-570-x/2024001/section2-eng.htm#a4> (e.g., Chart 3.1, 2015-2023) | **✖️** | **✅** |
| Public Health Agency of Canada (2025) Mental health of youth and young adults. [https://health-infobase.canada.ca/mental-health/youth-young-adults/technical-notes.html](https://can01.safelinks.protection.outlook.com/?url=https%3A%2F%2Fhealth-infobase.canada.ca%2Fmental-health%2Fyouth-young-adults%2Ftechnical-notes.html&data=05%7C02%7Calexandra.blair%40phac-aspc.gc.ca%7C1dca37610a154cdf2f9708de6dbf42ed%7C42fd9015de4d4223a368baeacab48927%7C0%7C0%7C639068865422865371%7CUnknown%7CTWFpbGZsb3d8eyJFbXB0eU1hcGkiOnRydWUsIlYiOiIwLjAuMDAwMCIsIlAiOiJXaW4zMiIsIkFOIjoiTWFpbCIsIldUIjoyfQ%3D%3D%7C0%7C%7C%7C&sdata=w44BaQ6kiaxKILBMugCmg99drxavEvsMxH1kpYGjh08%3D&reserved=0) (e.g., 2015 to 2022) - potential mode effects of 2022 cycle acknowledged in Technical Notes. | **✖️** | **✅** |
| McKinnon, B., Jahan, R., Mazza, J. (2025) Social inequalities in youth mental health in Canada, 2007–2022: a population-based repeated cross-sectional study [Social inequalities in youth mental health in Canada, 2007–2022: a population-based repeated cross-sectional study \| Social Psychiatry and Psychiatric Epidemiology \| Springer Nature Link](https://link.springer.com/article/10.1007/s00127-025-02813-7) | **✅** | **✅** |
| Public Health Agency of Canada (2024) Inequalities in mental health, well-being and wellness in Canada [Report - Inequalities in mental health, well-being and wellness in Canada \| Public Health Infobase - Public Health Agency of Canada](https://health-infobase.canada.ca/mental-health/inequalities/report.html) (e.g., 2007-2022) | **✅** | **✅** |
| Public Health Agency of Canada, Centre for Surveillance and Applied Research (2024). Positive Mental Health Surveillance Indicator Framework, adults (18 years of age and older), Canada, 2024 Edition. Ottawa (ON): Public Health Agency of Canada.  [Positive Mental Health Surveillance Indicator Framework (PMHSIF) — Canada.ca](https://health-infobase.canada.ca/positive-mental-health/data-tool/) (e.g., “Trend” Data Breakdown) | **✖️** | **✅** |
| Statistics Canada (2024) Health Reports: Trends in household food insecurity from the Canadian Community Health Survey, 2017 to 2022. [Trends in household food insecurity from the Canadian Community Health Survey, 2017 to 2022](https://www150.statcan.gc.ca/n1/pub/82-003-x/2024010/article/00002-eng.htm) | **✖️** | **✅** |
| British Columbia Centre for Disease Control – BC CDC (2026) BC Observatory for Population & Public Health: Canadian Community Health Survey (CCHS) Dashboard. [Canadian Community Health Survey (CCHS) Dashboard](https://www.bccdc.ca/health-professionals/data-reports/health-status/canadian-community-health-survey-dashboard#:~:text=Methodology:%20For%20a%20detailed%20description,the%20CCHS%20PUMF%20User%20Guides) | **✅** | **✖️** |
| Woolley, F. (2021). What Happened to the Immigrant Life Satisfaction Gap? Canadian Public Policy, 47(4), 510-522.: [https://muse.jhu.edu/pub/50/article/837960/pdf  .](https://muse.jhu.edu/pub/50/article/837960/pdf  . ) | **✅** | **✖️** |
| Su, Y.et al. (2022) Trends and patterns of life satisfaction and its relationship with social support in Canada, 2009 to 2018. Sci Rep 12, 9720. <https://doi.org/10.1038/s41598-022-13794-x> | **✅** | **✖️** |
| Lytvyak, E., Straube, S., Modi, R., & Lee, K. K. (2022). Trends in obesity across Canada from 2005 to 2018: a consecutive cross-sectional population-based study. *Canadian Medical Association Open Access Journal*, *10*(2), E439-E449. DOI: <https://doi.org/10.9778/cmajo.20210205> | **✅** | **✖️** |
| Su, Y.. et al. (2022) Trends and patterns of life satisfaction and its relationship with social support in Canada, 2009 to 2018. Sci Rep 12, 9720. <https://doi.org/10.1038/s41598-022-13794-x> | **✅** | **✖️** |
| Su, Y., D’Arcy, C., Li, M., & Meng, X. (2022). Determinants of Life Satisfaction and Self-Perceived Health in Nationally Representative Population-Based Samples, Canada, 2009 to 2018. Applied Research in Quality of Life, 17(6), 3285-3310. <https://link.springer.com/article/10.1007/s11482-022-10065-9> | **✅** | **✖️** |
| Dobson, K. G., Vigod, S. N., Mustard, C., & Smith, P. M. (2020). Trends in the prevalence of depression and anxiety disorders among working-age Canadian adults between 2000 and 2016. Health Reports, 31(12), 12-23. <https://statics.teams.cdn.office.net/evergreen-assets/safelinks/1/atp-safelinks.html> | **✅** | **✖️** |
| Salway, T., Rich et al. (2022). Preventable mortality among sexual minority Canadians. SSM-Population Health, 20, 101276. <https://www.sciencedirect.com/science/article/pii/S2352827322002555?via%3Dihub> | **✅** | **✖️** |
| Men, F., Gundersen, C., Urquia, M. L., & Tarasuk, V. (2020). Association between household food insecurity and mortality in Canada: a population-based retrospective cohort study. CMAJ, 192(3), E53-E60. <https://www.cmaj.ca/content/192/3/E53.short> | **✅** | **✖️** |
| Gagné, T., Lapalme, J., Ghenadenik, A. E., OLoughlin, J. L., & Frohlich, K. (2021). Socioeconomic inequalities in secondhand smoke exposure before, during and after implementation of Quebec’s 2015 ‘An Act to Bolster Tobacco Control’. Tobacco Control, 30(e2), e128-e137. <https://tobaccocontrol.bmj.com/content/30/e2/e128.abstract> | **✅** | **✖️** |
| Olstad, D. L., Nejatinamini, S., Victorino, C., Kirkpatrick, S. I., Minaker, L. M., & McLaren, L. (2021). Trends in socioeconomic inequities in diet quality between 2004 and 2015 among a nationally representative sample of children in Canada. The Journal of Nutrition, 151(12), 3781-3794. <https://www.sciencedirect.com/science/article/pii/S0022316622004485> | **✅** | **✖️** |
| Anderson, L. N., Islam, R., & Sweetman, A. (2025). Trends in obesity defined by body mass index among adults before and during the COVID-19 pandemic: a repeated cross-sectional study of the 2009–2023 Canadian Community Heath Surveys. *CMAJ*, *197*(25), E708-E718. <https://www.cmaj.ca/content/197/25/E708.short> | **✅** | **✅** |

**eTable 3:** A non-exhaustive snapshot of approved Statistics Canada Research Data Centre projects using CCHS cycles before and after the 2015 and/or 2022 redesigns

| **Research Projects** | **Combines cycles before and after 2015?** | **Combines cycles before and after 2022?** |
| --- | --- | --- |
| 2025-11-24 - Predictors of Sexual Orientation and Gender Identity Item Nonresponse in the Canadian Community Health Survey (CCHS), 2015–2024 Todd Coleman | **✖️** | **✅** |
| 2022-01-26 - Examining temporal trends in the co-occurrence of substance use and psychological distress among Canadians from 2003 to 2018 (Jillian Halladay) | **✅** | **✖️** |
| 2022-01-24 - Tendances dans la prévalence de la suicidalité au Canada de 2010 à 2021 chez les adolescents de 12-17 ans (Marie-Claude Geoffroy) | **✅** | **✖️** |
| 2020-03-13 - Trends in maternal influenza vaccination across Canada from 2000 to 2018 (Natalie Visca Scime) | **✅** | **✖️** |
| 2020-03-05 - Trends in mental health, alcohol, and smoking-related disparities between sexual minority and heterosexual adults in Canada, 2003-2018 (Travis James Salway) | **✅** | **✖️** |
| Details on these RDC projects can be found here: <https://www.statcan.gc.ca/en/microdata/data-centres/data/projects#wb-auto-2> | | |

**eTable 4: Indicator provincial and territorial data coverage across CCHS cycles**

| **Indicator** | **2007 to 2024** |
| --- | --- |
| Anxiety disorder | 2007-2022: all provinces and territories  2023: Only available for Nova Scotia, Quebec, Ontario, Manitoba, and Saskatchewan.  2024: all provinces (no Territory data). |
| Asthma | 2007-2020: all provinces and territories.  *2021, 2022, 2023, 2024: No data are available.* |
| Arthritis | 2007-2020: all provinces and territories.  2021: all provinces (no Territory data).  2022: all provinces and territories.  2023-2024: all provinces (no Territory data). |
| Community belonging (strong / very strong) | 2007-2020: all provinces and territories.  2021: all provinces (no Territory data).  2022: all provinces and territories.  2023-2024: all provinces (no Territory data). |
| COPD (Chronic obstructive pulmonary disorder) | 2009-2020: all provinces and territories.  *2021, 2022, 2023, 2024: No data are available.* |
| Diabetes (excluding gestational diabetes) | 2007-2020: all provinces and territories.  2021: all provinces (no Territory data).  2022: all provinces and territories.  2023-2024: all provinces (no Territory data). |
| Diabetes (including gestational diabetes) | 2007-2020: all provinces and territories.  2021: all provinces (no Territory data).  2022: all provinces and territories.  2023-2024: all provinces (no Territory data). |
| Food insecurity (moderate / severe) | 2007-2012: All provinces and territories.  2013-2014: All provinces and territories except Newfoundland and Labrador, Manitoba, Quebec, and Yukon.  2015-2016: All provinces and territories except Newfoundland and Labrador, Ontario, and Yukon.  2019-2020: All provinces and territories except British Columbia.  2021: All provinces (no Territory data).  2022: All provinces and territories.  2023-2024: All provinces (no Territory data). |
| Heart disease | 2007-2020: all provinces and territories.  2021: all provinces (no Territory data).  2022: all provinces and territories.  2023-2024: all provinces (no Territory data). |
| Hypertension | 2007-2020: all provinces and territories.  2021: all provinces (no Territory data).  2022: all provinces and territories.  2023-2024: all provinces (no Territory data). |
| Life satisfaction (satisfied / very satisfied) | 2009-2020: all provinces and territories.  2021: all provinces (no Territory data).  2022: all provinces and territories.  2023-2024: all provinces (no Territory data). |
| Life stress (quite / very stressful) | 2007-2020: all provinces and territories.  2021: all provinces (no Territory data).  2022: all provinces and territories.  2023-2024: all provinces (no Territory data). |
| Mood disorder | 2007-2020: all provinces and territories.  2021: all provinces (no Territory data).  2022: all provinces and territories.  2023-2024: all provinces (no Territory data). |
| Obesity | 2007-2020: all provinces and territories.  2021: all provinces (no Territory data).  2022: all provinces and territories.  2023-2024: all provinces (no Territory data). |
| Second-hand smoke exposure (home) | 2007-2016: all provinces and territories.  2017-2018: only data from Prince Edward Island, New Brunswick and Quebec.  2019-2020: only data from Prince Edward Island, New Brunswick and Quebec.  *2021, 2022, 2023, 2024: No data are available.* |
| Self-rated health (fair / poor) | 2007-2020: all provinces and territories.  2021: all provinces (no Territory data).  2022: all provinces and territories.  2023-2024: all provinces (no Territory data). |
| Self-rated health (very good / excellent) | 2007-2020: all provinces and territories.  2021: all provinces (no Territory data).  2022: all provinces and territories.  2023-2024: all provinces (no Territory data). |
| Self-rated mental health (fair / poor) | 2007-2020: all provinces and territories.  2021: all provinces (no Territory data).  2022: all provinces and territories.  2023-2024: all provinces (no Territory data). |
| Self-rated mental health (very good / excellent) | 2007-2020: all provinces and territories.  2021: all provinces (no Territory data).  2022: all provinces and territories.  2023-2024: all provinces (no Territory data). |
| Smoking, daily or occasionally in past 30 days | 2007-2020: all provinces and territories.  2021: all provinces (no Territory data).  2022: all provinces and territories.  2023-2024: all provinces (no Territory data). |

**eTable 5:** Regression models used for each of the decision-tree criteria, and corresponding R code

| **Percentage point change: Model** | **R code** |
| --- | --- |
| Generalized linear model (GLM) with logit link:  logit{Pr(Yi =1)}=β0 +β1 Time_i_  Where:  logit(p)=log(p / (1−p))  And where **Y** = 1 indicates the event (indicator) presence (0 = non-event), and **Time** values are {0 = pre-redesign period, 1 = post-redesign period}.  Predicted prevalence estimates on the risk scale are obtained by:  P_hat0 =Pr(Y=1∣T=0)  P_hat1 = Pr(Y=1∣T=1)  Percentage point change is obtained by:  P_hat1 – Phat0 | **For every indicator var, after setting data_path (file export location):**  *#Survey design set-up*  des_1415 <-svrepdesign (id=~1,  weights= ~FWGT,  repweights = "BSW[[:digit:]]+$",  type = "bootstrap",  combined.weights = TRUE,  mse = TRUE,  data=data1415)  *#Model function:*  fit_margins_export1415 <- function(var, time_var) {    *# (1) Build formula: var ~ time_var*  fml <- as.formula(paste(var, "~", time_var))    *# (2) Fit weighted logistic model*  mod <- svyglm(  formula = fml,  design = des_1415,  family = binomial(link = "logit")  )    *# (3) Margins (Average Marginal Effect [AME] of time on response scale)*    m <- margins(mod,  variables = time_var,  type = "response")    m_sum <- as.data.frame(summary(m))    *# (4) Add metadata to output*  m_sum$Outcome <- var  m_sum$Period <- "2014 vs. 2015"  m_sum$Lifecourse <- "Adults (18+)"  m_sum$Geography <- "Canada”  m_sum$Sex <- "Both sexes"  m_sum$Model <- "logit margins, weighted”  m_sum$Data <- "Average pp change in prevalence (APPC) - values already x100"    *#APPC = Average percentage point change*  m_sum$APPC<-round(m_sum$AME*100, 3)  *#95% Confidence interval:*  m_sum$APPC_LB<-round(  m_sum$lower*100, 3)  m_sum$APPC_UB<-round(m_sum$upper*100, 3)    m_sum <- dplyr::select(m_sum, Data, Geography, Outcome, Period, Lifecourse, Sex, APPC, APPC_LB, APPC_UB, p, Model)    *#(5) Export*  write_xlsx(m_sum,  paste0(data_path, "/Average_pp_change_2014v2015_", var, ".xlsx"))    return(m_sum)  }  *#End of function.*  *#Run the model for Indicator1, and Time {0 = pre, 1 = post)*  fit_margins_export1415(var = "Indicator1”, time_var = "time") |
| **Predicted prevalence for the post-redesign period: Model** | **R code** |
| Generalized linear model (GLM) with logit link:  logit{Pr(Yi =1)}=β0 +β1 TimeIndex_i_  Where:  logit(p)=log(p / (1−p ))  And where **Y** = 1 indicates the event (indicator) presence (0 = non-event), and **TimeIndex** values are, for example, {1 for 2007-2009, 2 for 2009-2010, 3 for 2011-2012, 4 for 2013-2014}  Predicted probability at **TimeIndex*** (e.g. 5 = for 2015-2016) is:  P_hat(TimeIndex*) =  =Pr(Y=1∣TimeIndex = TimeIndex* or 5)  P_hat(TimeIndex*) =  exp(β0 +β1 TimeIndex*) / (1+exp(β0 +β1 TimeIndex*)  Where P_hat(TimeIndex*) is the extrapolated model-projected prevalence.  **We repeat this model for 2022** using **TimeIndex**, where values are set to {1 for 2015-2016, 2 2 for 2017-2018, 3 for 2019-2021}, predicting values for for **TimeIndex* = 4 (2022).** | **For every indicator var, after setting data_path (file export location):**  *#Survey design set-up*  des_0914 <-svrepdesign (id=~1,  weights= ~FWGT,  repweights = "BSW[[:digit:]]+$",  type = "bootstrap",  combined.weights = TRUE,  mse = TRUE,  data=data0914)  *#Model function:*  predict_function15<-function(indicator, time_var) {    *# (1) Build formula: indicator ~ time_var*  fml <- as.formula(paste(indicator, "~", time_var))    *# (2) Fit weighted logistic model*  fit <- svyglm(  formula = fml,  design = des_0914,  family = binomial(link = "logit")  )    # (3) Create a prediction dataframe that only contains the value we want of time  *#2015 would fall on time = 5 (based on TimeIndex logic)*  newdat <- data.frame(time = 5)    *# (4) Pass the new data to the predict() function:*  *# Get prediction on the link (logit) scale for clean CI, then transform*    pred_link <- predict(fit, newdata = newdat, type = "link", se.fit = TRUE)    *# (5) Extract predicted prevalence and its 95% CI*    *# point estimate on the link (logit) scale*  eta <- as.numeric(pred_link)  *# standard error on the link scale*  se <- sqrt(attr(pred_link, "var"))  *#95% CI bounds*  lwr <- eta - 1.96 * se  upr <- eta + 1.96 * se    *#Prepare export dataframe*  df <- data.frame(  Indicator = indicator,  time = newdat$time,  *#Transform eta into a probability using the logistic (inverse-logit) function:*  pred_prob = round(plogis(eta)*100, 3),  ci_lower = round(plogis(lwr)*100, 3),  ci_upper = round(plogis(upr)*100, 3)  )    *#(6) Export*  write_xlsx(df,  paste0(data_path, "/Model-based_prediction_2015_", indicator, ".xlsx"))    return(df)  }  *#End of function*  *#Run the model for Indicator1, and Time {1,2,3,4}*  predict_function15(indicator = "Indicator1”, time_var = "time") |

**eTable 6:** Crude prevalence of all indicators, across all cycles, from 2007-2008 to 2024 among adults, aged 18 years and older)

| **Indicator** | **2007-2008** | **2009-2010** | **2011-2012** | **2013-2014** | **2015-2016** | **2017-2018** | **2019-2021** | **2022** | **2023** (Provinces only) | **2024** (Provinces only) |
| --- | --- | --- | --- | --- | --- | --- | --- | --- | --- | --- |
| Anxiety disorder | 5.71 (5.49, 5.93) | 5.31 (5.08, 5.54) | 6.56 (6.28, 6.85) | 7.05 (6.78, 7.33) | 8.21 (7.92, 8.51) | 9.09 (8.8, 9.37) | 10.06 (9.73, 10.39) | 15.08 (14.56, 15.6) | 15.58 (14.83, 16.33) | 16.66 (16.02, 17.3) |
| Asthma | 7.89 (7.64, 8.14) | 8.07 (7.77, 8.37) | 8.14 (7.86, 8.43) | 7.92 (7.65, 8.2) | 8.39 (8.07, 8.71) |  |  |  |  |  |
| Arthritis | 16.74 (16.43, 17.04) | 16.69 (16.35, 17.02) | 17.07 (16.72, 17.43) | 16.97 (16.63, 17.31) | 21.27 (20.9, 21.65) | 19.75 (19.37, 20.13) | 20.28 (19.96, 20.6) | 20.57 (20.08, 21.07) | 20.93 (20.39, 21.47) | 21.23 (20.68, 21.77) |
| Community belonging (strong / very strong) | 63.37 (62.85, 63.9) | 64.1 (63.55, 64.64) | 64.23 (63.67, 64.8) | 65.05 (64.51, 65.59) | 67.11 (66.57, 67.64) | 67.59 (67.08, 68.1) | 68.56 (68.05, 69.06) | 63.44 (62.68, 64.2) | 60.69 (59.86, 61.51) | 61.21 (60.35, 62.07) |
| COPD (Chronic obstructive pulmonary disorder) | *NA* | 4.24 (4.04, 4.44) | 4.22 (4, 4.45) | 4.13 (3.92, 4.34) | 4.14 (3.92, 4.35) |  |  |  |  |  |
| Diabetes (excluding gestational diabetes) | 6.37 (6.16, 6.58) | 6.62 (6.38, 6.86) | 6.86 (6.6, 7.13) | 7.09 (6.84, 7.34) | 7.31 (7.06, 7.57) | 7.57 (7.31, 7.82) | 7.83 (7.61, 8.06) | 8.24 (7.88, 8.61) | 7.9 (7.54, 8.25) | 8.66 (8.27, 9.05) |
| Diabetes (including gestational diabetes) | 6.46 (6.24, 6.68) | 6.68 (6.44, 6.93) | 6.94 (6.67, 7.2) | 7.18 (6.93, 7.43) | 7.41 (7.15, 7.67) | 7.71 (7.45, 7.97) | 7.96 (7.73, 8.19) | 8.36 (7.99, 8.72) | 8.01 (7.65, 8.36) | 8.8 (8.41, 9.19) |
| Food insecurity (moderate / severe) | 6.92 (6.6, 7.23) | 6.9 (6.6, 7.2) | 7.56 (7.23, 7.89) | 7.27 (6.91, 7.62) | 7.54 (7.18, 7.9) | 8.07 (7.74, 8.4) | 6.2 (5.93, 6.48) | 10.91 (10.41, 11.4) | 12.57 (12.01, 13.13) | 13.75 (13.11, 14.39) |
| Heart disease | 5.28 (5.09, 5.46) | 5.21 (5.01, 5.4) | 5.34 (5.13, 5.56) | 5.21 (5.01, 5.41) | 4.94 (4.74, 5.14) | 4.89 (4.7, 5.08) | 5.12 (4.95, 5.29) | 5.4 (5.13, 5.68) | 5.37 (5.09, 5.66) | 5.54 (5.23, 5.85) |
| Hypertension | 18.88 (18.53, 19.24) | 19.51 (19.12, 19.89) | 20.1 (19.75, 20.46) | 20.3 (19.95, 20.65) | 21.2 (20.84, 21.56) | 21.61 (21.25, 21.97) | 21.68 (21.36, 22.01) | 22.57 (22.08, 23.06) | 22.3 (21.77, 22.83) | 23.47 (22.9, 24.04) |
| Life satisfaction (satisfied / very satisfied) | *NA* | 91.67 (91.37, 91.98) | 91.99 (91.67, 92.3) | 91.64 (91.31, 91.98) | 92.49 (92.21, 92.77) | 92.77 (92.48, 93.05) | 92.73 (92.47, 92.99) | 87.28 (86.76, 87.8) | 85.52 (84.91, 86.13) | 84.08 (83.39, 84.77) |
| Life stress (quite / very stressful) | 22.82 (22.37, 23.28) | 23.82 (23.36, 24.27) | 23.46 (22.98, 23.93) | 23.17 (22.66, 23.67) | 22.18 (21.72, 22.64) | 21.99 (21.54, 22.44) | 21.26 (20.84, 21.67) | 22.25 (21.62, 22.87) | 22.23 (21.57, 22.89) | 22.67 (21.89, 23.45) |
| Mood disorder | 7.05 (6.82, 7.28) | 6.98 (6.71, 7.25) | 7.56 (7.29, 7.83) | 8.1 (7.81, 8.4) | 8.48 (8.19, 8.77) | 9.17 (8.84, 9.51) | 9.72 (9.39, 10.04) | 12.02 (11.54, 12.51) | 13.65 (13.08, 14.23) | 13.63 (13.02, 14.24) |
| Obesity | 23.23 (22.84, 23.62) | 23.84 (23.38, 24.3) | 24.79 (24.32, 25.26) | 26.14 (25.65, 26.63) | 26.45 (25.98, 26.93) | 26.95 (26.45, 27.46) | 28.64 (28.17, 29.11) | 30.32 (29.63, 31.02) | 30.35 (29.59, 31.1) | 31.26 (30.43, 32.09) |
| Second-hand smoke exposure (home) | 5.76 (5.51, 6.01) | 5.1 (4.83, 5.37) | 4.37 (4.1, 4.64) | 3.63 (3.41, 3.84) | 3.12 (2.90, 3.34) |  |  |  |  |  |
| Self-rated health (fair / poor) | 12.13 (11.83, 12.42) | 12.03 (11.71, 12.35) | 11.94 (11.57, 12.3) | 11.89 (11.57, 12.21) | 11.58 (11.25, 11.92) | 11.71 (11.39, 12.03) | 11.52 (11.23, 11.81) | 14.56 (14.04, 15.08) | 14.52 (13.98, 15.05) | 14.92 (14.31, 15.53) |
| Self-rated health (very good / excellent) | 58.29 (57.79, 58.79) | 59.4 (58.89, 59.91) | 59 (58.42, 59.57) | 58.4 (57.89, 58.91) | 60.49 (59.97, 61.02) | 59.81 (59.28, 60.35) | 60.03 (59.54, 60.51) | 53.74 (52.96, 54.51) | 52.2 (51.39, 53.01) | 52.11 (51.27, 52.96) |
| Self-rated mental health (fair / poor) | 5.08 (4.86, 5.3) | 5.43 (5.19, 5.67) | 5.91 (5.66, 6.17) | 6.42 (6.15, 6.69) | 6.6 (6.32, 6.88) | 7.49 (7.19, 7.79) | 9.91 (9.58, 10.25) | 14.45 (13.89, 15.02) | 14.97 (14.37, 15.57) | 15.12 (14.48, 15.75) |
| Self-rated mental health (very good / excellent) | 74.14 (73.7, 74.59) | 73.57 (73.09, 74.06) | 71.68 (71.13, 72.24) | 70.76 (70.24, 71.28) | 71.15 (70.64, 71.66) | 69 (68.51, 69.49) | 63.49 (62.97, 64) | 54.61 (53.81, 55.42) | 53.86 (53.05, 54.66) | 53.72 (52.84, 54.59) |
| Smoking, daily or occasionally in past 30 days | 21.88 (21.45, 22.3) | 20.47 (20.05, 20.89) | 20.02 (19.56, 20.49) | 18.49 (18.04, 18.95) | 17.13 (16.71, 17.54) | 15.78 (15.36, 16.2) | 12.81 (12.47, 13.16) | 12.47 (11.98, 12.95) | 11.2 (10.7, 11.7) | 11.1 (10.6, 11.7) |

**eTable 7:** Micro-trend sensitivity analyses of crude prevalence of select indicators, in 2020-2021, 2022 (pre-redesign), 2022 (post-redesign), 2023-2024 among adults.

| **Indicator** | **2020** | **2021**  (Prov. only) | **2022**  (pre-redesign) | **2022**  (post-redesign | **2023**  (Prov. only) | **2024**  (Prov. only) |
| --- | --- | --- | --- | --- | --- | --- |
| Food insecurity (moderate / severe) | 6.02 (5.52, 6.53) | 5.67 (5.21, 6.12) | 6.02 (4.63, 7.41) | 10.91 (10.41, 11.4) | 12.57 (12.01, 13.13) | 13.75 (13.11, 14.39) |
| *Change from previous year* | - | - 0.4 PP | + 0.4 PP | + 4.9 PP |  |  |
| Self-rated health (very good / excellent) | 61.3 (60.4, 62.2) | 58.6 (57.7, 59.5) | 60.5 (57.7, 63.4) | 53.7 (53.0, 54.5) | 52.2 (51.39, 53.01) | 52.11 (51.27, 52.96) |
| *Change from previous year* | - | -2.7 PP | +1.9 | -6.8 PP |  |  |
| Self-rated mental health (fair / poor) | 9.80 (9.20, 10.4) | 11.7 (11.1, 12.3) | 11.7 (9.76, 13.7) | 14.45 (13.89, 15.02) | 14.97 (14.37, 15.57) | 15.12 (14.48, 15.75) |
| *Change from previous year* | - | +1.9 PP | 0 PP | +2.8 |  |  |
| Self-rated mental health (very good / excellent) | 64.9 (64.0, 65.9) | 59.2 (58.2, 60.1) | 56.3 (53.3, 59.3) | 54.6 (53.8, 55.4) | 53.86 (53.05, 54.66) | 53.72 (52.84, 54.59) |
| *Change from previous year* | - | -5.7 PP | -2.9 PP | -1.7 PP |  |  |
| **Note:** These were indicators for which all decision-tree thresholds were crossed, in the 2022 CCHS redesign sensitivity analyses; the pre-redesign 2022 data are obtained from the CCHS 2021. “Prov.” refers to Province. Cycles 2020 and 2022 contained data from provinces and territories. “PP” refers to percentage point change, compared to the preceding period / column. | | | | | | |

**eTable 8:** Examples of proposed caution notes for HIRI data users, for indicators that met or did not meet all decision-free flags

| **Indicator** | **Decision-tree analyses results** | **HIRI Decision & Example of a caution note**  that would accompany any released data |
| --- | --- | --- |
| Self-rated health (very good / excellent) | **Absolute change > 5-PP?**  Yes  **Relative change > 10%?**  Yes  **No overlap between predicted and observed post-redesign prevalence?**  Yes  **Literature search corroborates observed trend?**  Mixed findings: corroborating evidence of COVID-19-related declines in health, but alternative data sources do not depict identical patterns of decrease. | **Decision:**  Given the mixed findings, continue to combine data pre- and post-2022 for long-term trends reporting, but provide a caution note.  **Caution note:**  “The Canadian Community Health Survey (CCHS) underwent major redesigns in 2015 and 2022. As a result, Statistics Canada recommends that caution should be used when comparing data between various cycles pre- and post-redesigns. Sensitivity analyses were conducted to assess the potential influence of these methodological changes on trends over time. Analyses suggest that although it is plausible that self-rated health declined in the COVID-19 period, the 2022 redesign likely influenced the increased magnitude of the observed decline immediately before and after 2022. Long-term inequality trends should therefore be interpreted with caution.” |
| Self-rated mental health (very good / excellent)  Self-rated mental health (fair / poor) | **Absolute change > 5-PP?**  Yes  **Relative change > 10%?**  Yes  **No overlap between predicted and observed post-redesign prevalence?**  Yes  **Literature search corroborates observed trend?**  Mixed findings: corroborating evidence of COVID-19-related declines in mental health, but alternative data sources do not depict identical patterns of decrease. | **Decision:**  Given the mixed findings, continue to combine data pre- and post-2022 for long-term trends reporting, but provide a caution note.  **Caution note:**  “The Canadian Community Health Survey (CCHS) underwent major redesigns in 2015 and 2022. As a result, Statistics Canada recommends that caution should be used when comparing data between various cycles pre- and post-redesigns. Sensitivity analyses were conducted to assess the potential influence of these methodological changes on trends over time. Analyses suggest a decline in self￼rated mental health is observable in Canada between 2007 and 2024; however, the 2022 redesign may have influenced the increased magnitude of the observed decline between the pre￼ and post￼2022 periods. Long-term inequality trends should therefore be interpreted with caution.” |
| Food insecurity (moderate / severe) | **Absolute change > 2.5-PP?**  Yes  **Relative change > 10%?**  Yes  **No overlap between predicted and observed post-redesign prevalence?**  Yes  **Literature search corroborates observed trend?**  Yes | **Decision:**  Given the corroborative findings, continue to combine data pre- and post-2022 for long-term trends reporting, but provide a caution note.  **Caution note:**  “The Canadian Community Health Survey (CCHS) underwent major redesigns in 2015 and 2022. As a result, Statistics Canada recommends that caution should be used when comparing data between various cycles pre- and post-redesigns. Sensitivity analyses were conducted to assess the potential influence of these methodological changes on trends over time. Research suggests that that the observed increase in food insecurity for Canada overall, as of 2022, may be due in part to rising food price inflation. However, residual effects of the redesign cannot be ruled out. See: Trends in household food insecurity from the Canadian Community Health Survey, 2017 to 2022: https://www150.statcan.gc.ca/n1/pub/82-003-x/2024010/article/00002-eng.htm” |
| Anxiety | **Absolute change > 5-PP?**  No  **Relative change > 10%?**  Yes  **No overlap between predicted and observed post-redesign prevalence?**  Yes  *No literature search performed, since not all criteria received a “yes” response.* | **Decision:**  As the three change criteria were not met, continue to combine data pre- and post-2022 for long-term trends reporting, but provide a caution note.  **Caution note:**  “The Canadian Community Health Survey (CCHS) underwent major redesigns in 2015 and 2022. As a result, Statistics Canada recommends that caution should be used when comparing data between various cycles pre- and post-redesigns. Sensitivity analyses were conducted to assess the potential influence of these methodological changes on trends over time. Overall, the analyses suggest limited impact on this indicator; however, residual effects cannot be ruled out.” |

Note: “HIRI” refers to the Public Health Agency of Canada’s Health Inequalities Reporting Initiative.
